# Epidemiological trends and healthcare disparities in onychomycosis: An analysis of the All of Us research program

**DOI:** 10.1371/journal.pone.0316681

**Published:** 2025-01-14

**Authors:** Aditya K. Gupta, Vasiliki Economopoulos, Tong Wang, Vincent Piguet

**Affiliations:** 1 Division of Dermatology, Department of Medicine, Temerty Faculty of Medicine, University of Toronto, Toronto, Ontario, Canada; 2 Mediprobe Research Inc., London, Ontario, Canada; 3 Department of Medical Biophysics, Schulich Scholl of Medicine and Dentistry, University of Western Ontario, London, Ontario, Canada; 4 Division of Dermatology, Department of Medicine, Women’s College Hospital, Toronto, Ontario, Canada; Gulu University, UGANDA

## Abstract

Onychomycosis is a common, difficult to treat nail disorder. Our objective was to explore disparities in current clinical management practices for onychomycosis in patients from underrepresented groups and with specific comorbidities. We conducted a cross-sectional study using the All of Us (AoU) research program. The AoU program gathers survey, and electronic health records from participants in the United States with the aim of increasing the representation of minorities groups in health research under the framework of precision medicine. We identified 18,763 onychomycosis patients (2017–2022) and compared the rates of diagnostic testing, prescription medications and surgical procedures. Younger patients were more likely to receive oral medications, while older patients were more likely to undergo surgical nail procedures. Patients with lower income and education, Black and Hispanic patients were less likely to receive testing to confirm diagnosis, and less likely to receive prescription medications (topical and/or oral) except in the case of fluconazole. Lower income and education were associated with a higher likelihood of debridement and trimming procedures, while Black and Hispanic patients were less likely to undergo these procedures. Patients with disabilities also received different treatments when compared to able-bodied individuals, being less likely to receive ciclopirox, efinaconazole and terbinafine, but more likely to undergo debridement and trimming procedures. There are clear differences in the management of onychomycosis in the different demographic and comorbid populations that we studied. Efforts to reduce these inequalities, such as expanded health coverage, reducing communication barriers and increasing patient and physician education are needed.

## Introduction

Onychomycosis is the most common nail disorder diagnosed in adults, with an incidence of 2–5% and up to 20% lifetime prevalence [1–4]. Cases are often difficult to treat and have a high risk of recurrence [2, 5]. Understanding the epidemiology of onychomycosis and variations in management strategies are essential for improving patient outcomes.

The difficulty in managing onychomycosis underlies rising comorbidity burdens amidst an aging population (e.g., polypharmacy); [6] for instance, the higher risk of onychomycosis in the geriatric population is compounded by diabetes and peripheral vascular disease that can increase infection risk through the secondary development of ulceration of the foot [7–9]. Furthermore, the demographic makeup of the dermatology patient population is ever-changing, which has led to an increase in healthcare disparities [10]. According to the 2023 National Healthcare Quality of Disparities Report [11], there has been a continued rise in the population of ethnic minorities—who often face additional barriers in accessing dermatology care—which now account for close to half of the U.S. population [12]. As well, social determinants of health play a significant role in the ability to access dermatology care [13].

In recognition of an increasingly diverse patient population including those with onychomycosis, we performed a cross-sectional study using the All of Us database hosted by the National Institute of Health as part of its research initiative in precision medicine. Previous studies by Albucker et al. and Moseley et al. have reported patient demographic risk factors as well as treatment trends of onychomycosis using the All of Us database [14, 15]. The aim of the present study was to explore potential disparities in the current management practices concerning diagnostic testing (i.e., culture, KOH direct microscopy, nucleic acid amplification) and treatment (i.e., topical and oral antifungals, surgical nail procedures).

## Materials and methods

The All of Us research program, which aims to provide diverse and inclusive health data was accessed for this work. All analyses were performed using the registered tier dataset version 7 available within the All of Us research program, which includes data collected from US participants between the summer of 2017 and July 1, 2022. This dataset includes electronic health records collected from health care providers for individuals that have volunteered to participate in the program. Additional information regarding the All of Us research program can be found in the indicated references [16–18].

Ethics approval was not required for this study as the AoU is a resource for which prior ethics approvals for the program were acquired by the NIH. Ethics agreements have been put in place with registered partner institutions on the access and use of the data. No patient identifiers were collected. All work was conducted in accordance with STROBE/RECORD (S1 File) and PLOS One human participants research guidelines (S2 File).

We accessed data with AoU between July 1, 2024 and September 1, 2024 and included only registered participants that had electronic health record data available, which included a total of 266,612 individuals. We accessed data from patients that had completed the survey questions of interest for demographic factors. To determine onychomycosis risk in participants with co-morbid conditions, we accessed data from all participants with the specific co-morbidity and then selected age- and sex- matched healthy controls from a cohort of participants without the co-morbidities of interest. The number of comorbid participants and the number of matched controls are listed in S1 Table. We then reviewed clinically confirmed onychomycosis cases and those with co-morbidities. We identified the diagnostic tests conducted as well as medical and surgical treatments offered, using the concept codes and IDs coded within the Observational Medical Outcomes Partnership (OMOP) framework.

Survey responses for demographic factors—including age at diagnosis of onychomycosis (or age at program intake for no onychomycosis), sex at birth, economic status, education, ethnicity/race and disability—were examined to identify any disparities between these factors on onychomycosis burden and management. The number of participants in each demographic group are listed in S2 Table.

We examined the following comorbid conditions and their potential impact on the burden of onychomycosis: type 2 diabetes mellitus (DM), obesity, tinea pedis, peripheral artery disease, peripheral venous insufficiency, peripheral neuropathy in the lower limbs, edema in the lower limbs, arthritis in the lower limbs, deformities of the lower limbs, patients undergoing hemodialysis, patients that have received a renal transplant, HIV positive patients, psoriasis, lupus erythematosus, and chronic liver disease.

The diagnostic testing rates (including fungal culture, presence of fungus by KOH preparation, and nucleic acid amplification tests), prescribing rates of topical (ciclopirox, efinaconazole, tavaborole) and oral (terbinafine, itraconazole and fluconazole) antifungals, and the rate of nail-related surgical procedures (nail avulsion, debridement of nail(s) and trimming of nail(s)), were determined for onychomycosis patients stratified by the demographic factors listed above.

Data in all graphs and tables are presented as percentages of participants where applicable in an effort to protect patient privacy in accordance with the All of Us research program mandate. Tables where the number of patients is less than 20 have a value of <20 shown to protect patient privacy where applicable. N-values for all groups are shown within the data shown in the supplementary tables S1 and S2 Tables. All error bars are shown as standard error. All concept ID codes used in this work are listed in S3 Table.

All analyses were performed within the Researcher Workbench environment using the SAS Studio application. Statistical analyses were performed using the Rao-Scott and Wald’s chi-square (χ^2^) tests. We calculated the unadjusted odds ratio (OR) for each comparison with a significance level α = 0.05. The Benjamini-Hochberg method was used to adjust for multiple comparisons with a false discovery rate (Q) of 0.1.

## Results

### Epidemiology of onychomycosis

**Fig 1:**
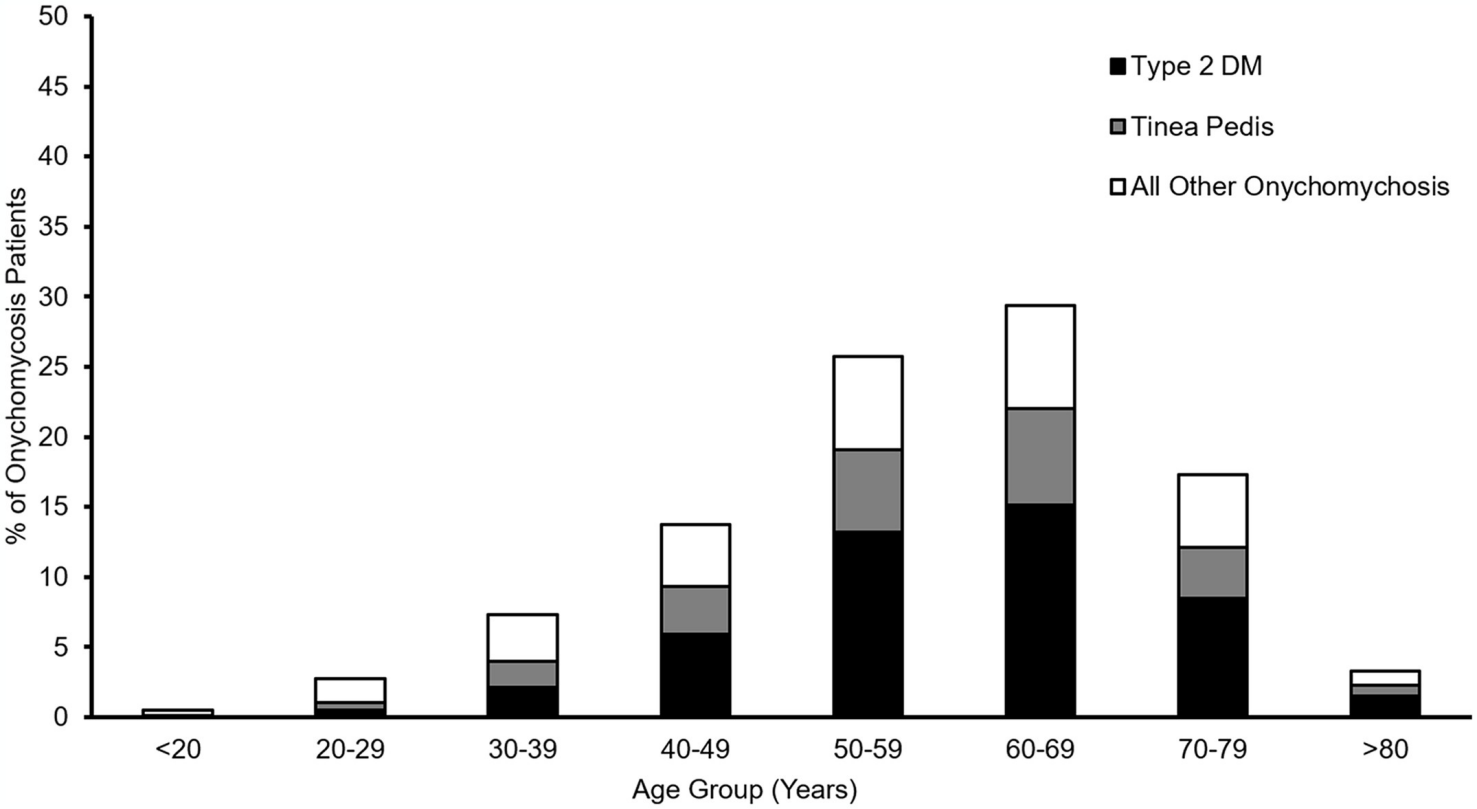
Age distribution of onychomycosis patients at first diagnosis. Black represents patients with comorbid type 2 DM. Grey represents patients with tinea pedis. White represents all other onychomycosis patients. Data are presented as percentages to meet AoU data presentation requirements.

We examined the age distribution of patients at initial diagnosis (Fig 1, n = 18,763); there was a higher burden of onychomycosis in 50-59- and 60-69-years old patients (p < 0.001, Rao-Scott), a substantial number of patients had a co-diagnosis of type 2 diabetes mellitus or tinea pedis. Lower income (less than $75,000/year) and lower education (less than Bachelor’s degree) were associated with an increased risk of onychomycosis (Economic: OR 1.20 (1.16–1.24), p < 0.001; Education: OR 1.09 (1.06–1.12), p <0.001). Patients of African American (Black) ethnicity/race had a significantly higher burden of onychomycosis (OR 1.25 (1.20–1.29), p <0.001) than the reference group (White patients).

**Fig 2:**
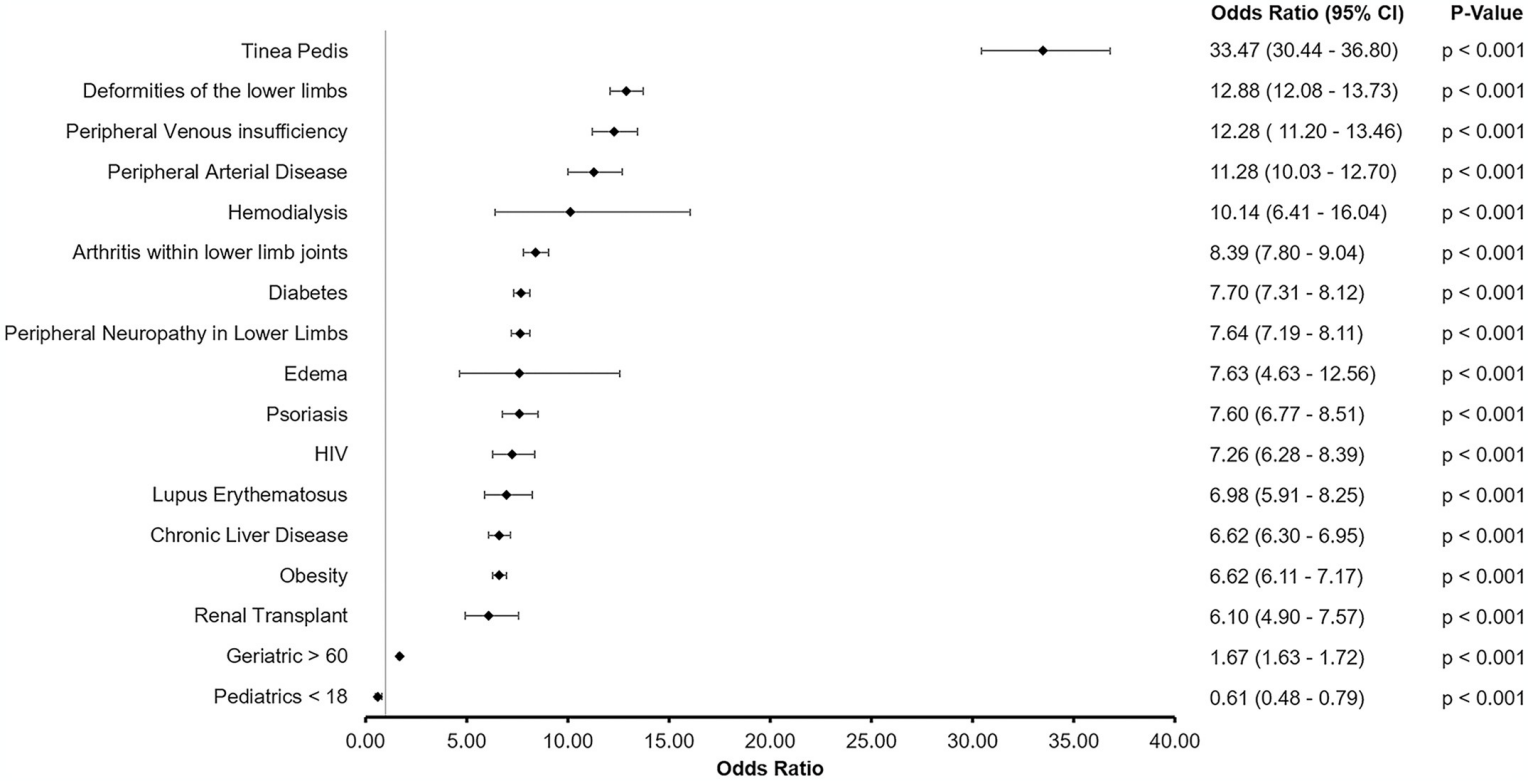
Odds ratio and p-value for likelihood of onychomycosis in comorbid participants compared to otherwise healthy control participants.

We examined the relationship between onychomycosis and comorbidity (Fig 2). All of the comorbidities examined showed a significantly increased rate of onychomycosis diagnoses; however, we found that the associated odds ratio was the highest for participants with onychomycosis that had co-existing tinea pedis (OR 33.47 (30.44–36.80), p < 0.001) followed by deformities of the lower limbs (OR 12.88 (12.08–13.73), p < 0.001), peripheral venous insufficiency (OR 12.28 (11.2–13.46), p < 0.001) and peripheral arterial disease (OR 11.28 (10.03–12.07), p <0.001).

### Diagnostic testing practices

Diagnostic testing rates were identified for each demographic group as well as for four comorbid conditions: tinea pedis, obesity, HIV and Type 2 diabetes mellitus (Table 1). Mycological testing to confirm the clinical diagnosis was more frequently conducted in patients that have an income greater than $75,000 USD/year (OR 1.54 (1.23–1.92), p < 0.001) and hold at least a bachelor’s degree (OR 1.79 (1.45–2.20), p < 0.001).

**Table 1:** Participants that received diagnostic testing by demographic factor and comorbidity.

	% of Participants That Received Testing	Odds Ratio	P-Value
*Geriatric*			
> 60 years (Ref)	1.95	Ref	
< 60 years	1.79	0.92 (0.74–1.14)	0.428
*Sex at Birth*			
Female (Ref)	2.36	Ref	
Male	1.75	0.74 (0.57–0.96)	0.022 *
*Income*			
< $75,000/year (Ref)	1.96	Ref	
> $75,000/year	3.01	1.54 (1.23–1.92)	<0.001 *
*Education*			
Some College, High School or Less	1.56	Ref	
Bachelor’s Degree or Higher	2.79	1.79 (1.45–2.20)	< 0.001 *
*Race/Ethnicity*			
Black	0.89	0.33 (0.23–0.46)	< 0.001 *
Hispanic	1.52	0.56 (0.41–0.77)	< 0.001 *
Other	2.05	0.75 (0.44–1.27)	0.232
White (Ref)	2.72	Ref	
*Disability*			
Disabled (Ref)	1.73	Ref	
Not Disabled	2.46	1.42 (0.87–2.30)	0.143
*Comorbid Conditions*			
Tinea Pedis	1.4	1.69 (1.14–2.49)	0.009 *
Obesity	1.3	1.56 (1.10–2.20)	0.007 *
HIV	0.7	0.81 (0.32–2.05)	0.634
Type 2 Diabetes	1.1	1.34 (0.94–1.92)	0.092
No Comorbidity	0.8	Ref	

Patients were less likely to undergo diagnostic testing to confirm the clinical diagnosis of onychomycosis if they were male (OR 0.74 (0.57–0.96), p = 0.022), Black (OR 0.33 (0.23–0.46), p < 0.001) or Hispanic (OR 0.56 (0.41–0.77), p < 0.001) (Table 1).

When examining diagnostic testing patterns in patients with comorbid conditions, patients with tinea pedis and obesity were more likely to have received testing (OR 1.69 (1.14–2.49), p = 0.009; OR 1.56 (1.10–2.20), p = 0.007; respectively) when compared to otherwise healthy patients.

### Medical prescription trends

**Fig 3:**
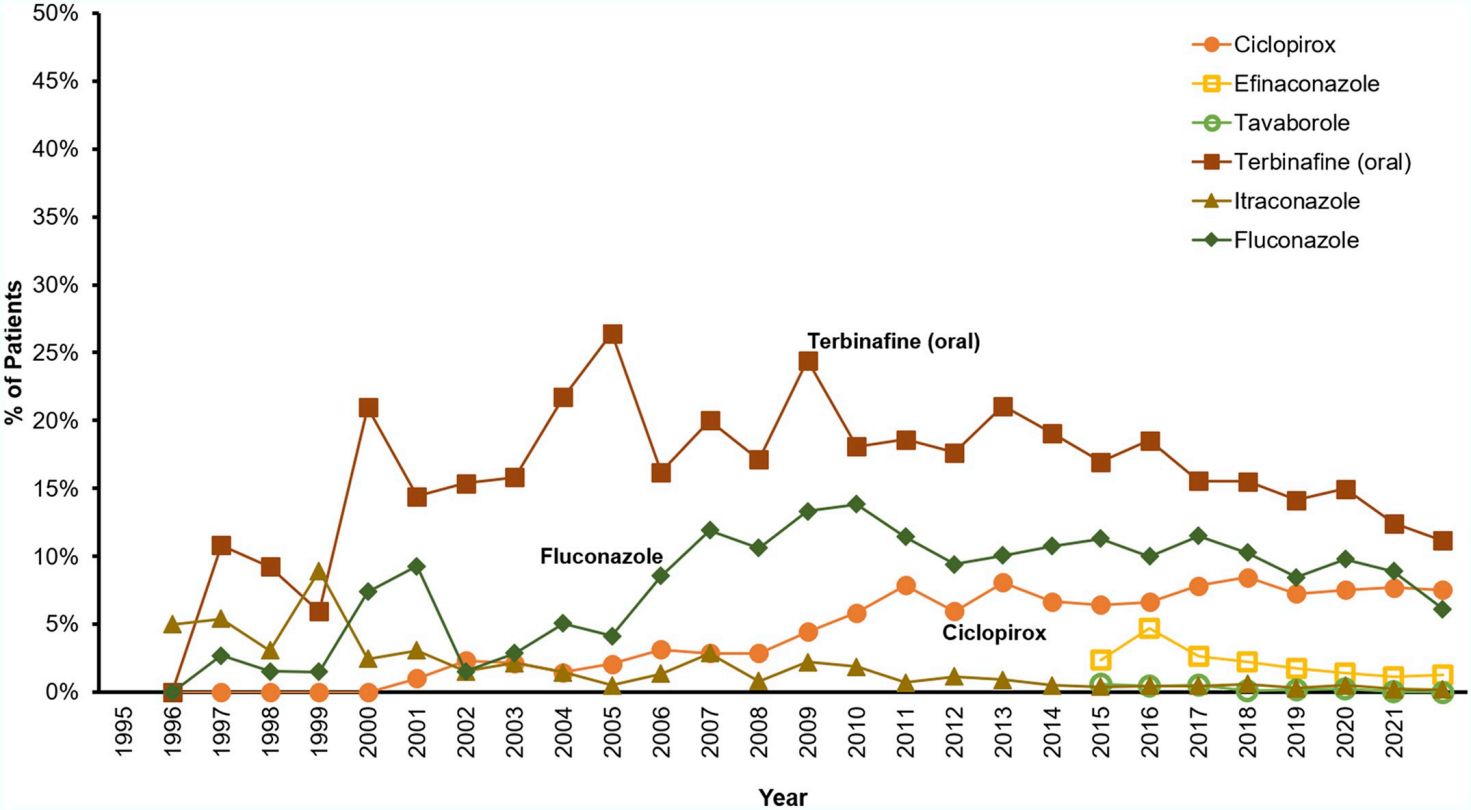
Percentage of onychomycosis patients receiving prescription medications per year.

**Fig 4:**
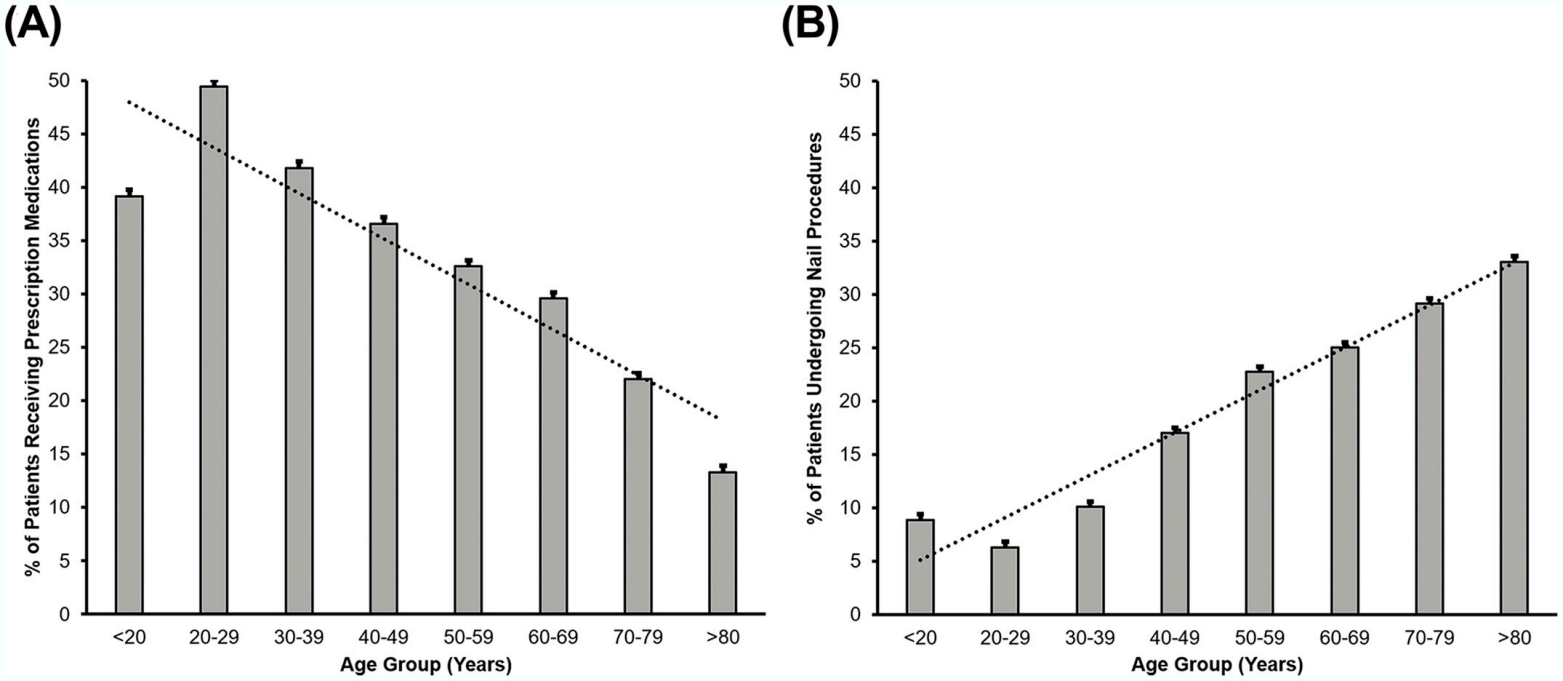
Age distribution of patients receiving (A) prescription medications and (B) undergoing nail related procedures.

Oral terbinafine, fluconazole and topical ciclopirox nail lacquer are prescribed more frequently than itraconazole, topical efinaconazole and topical tavaborole (Fig 3). The proportion patients receiving prescription medications decreases with age (p < 0.001, Rao-Scott) (Fig 4A).

Patients less than 60 years of age were more likely to be prescribed terbinafine (OR 1.70 (1.54–1.88), p < 0.001), itraconazole (OR 3.06 (1.77–5.27), p < 0.001) and fluconazole (OR 1.77 (1.56–2.00), p < 0.001) (Table 2). Males were less likely to be prescribed ciclopirox (OR 0.60 (0.52–0.70), p < 0.001), tavaborole (OR 0.29 (0.11–0.79), p = 0.009) and fluconazole (OR 0.27 (0.23–0.31), p < 0.001) compared to females. Those who made greater than $75,000 USD/year were more likely to be prescribed ciclopirox (OR 1.73 (1.51–1.97), p < 0.001), efinaconazole (OR 3.47 (2.66–4.54), p < 0.001), tavaborole (OR 5.36 (2.44–11.78), p < 0.001), terbinafine (OR 1.54 (1.40–1.69), p < 0.001) and itraconazole (OR 2.36 (1.60–3.50), p < 0.001), but were less likely to be prescribed fluconazole (OR 0.79 (0.69–0.90), p < 0.001).

**Table 2:** Odds Ratios and P-values for prescribed medications.

	Ciclopirox		Efinaconazole		Tavaborole		Terbinafine (oral)		Itraconazole		Fluconazole	
	OR (95% CI)	P-Value	OR (95% CI)	P-Value	OR (95% CI)	P-Value	OR (95% CI)	P-Value	OR (95% CI)	P-Value	OR (95% CI)	P-Value
*Geriatric*												
< 60 years VS > 60 years (Ref)	1.01 (0.89–1.14)	0.919	0.94 (0.72–1.24)	0.669	1.35 (0.60–3.01)	0.444	1.70 (1.54–1.88)	< 0.001 *	3.06 (1.77–5.27)	< 0.001 *	1.77 (1.56–2.00)	< 0.001 *
*Sex at Birth*												
Male Vs Female (Ref)	0.60 (0.52–0.70)	< 0.001 *	0.73 (0.54–0.98)	0.033	0.29 (0.11–0.79)	0.009 *	1.03 (0.93–1.14)	0.598	1.44 (0.93–2.24)	0.100	0.27 (0.23–0.31)	< 0.001 *
*Income*												
> $75,000/year VS < $75,000/year (Ref)	1.73 (1.51–1.97)	< 0.001 *	3.47 (2.66–4.54)	< 0.001 *	5.36 (2.44–11.78)	0.001 *	1.54 (1.40–1.69)	< 0.001 *	2.36 (1.60–3.50)	<0.001 *	0.79 (0.69–0.90)	<0.001 *
*Education*												
Bachelor’s Degree or Higher VS Some College, High School or Less (Ref)	1.64 (1.46–1.84)	< 0.001 *	3.07 (2.38–3.96)	< 0.001 *	3.05 (1.48–6.30)	0.004 *	1.36 (1.25–1.47)	< 0.001 *	2.08 (1.44–3.00)	<0.001 *	0.82 (0.73–0.91)	<0.001 *
*Race/Ethnicity*												
Black	0.71 (0.61–0.83)	< 0.001 *	0.38 (0.26–0.56)	< 0.001 *	0.09 (0.01–0.69)	< 0.001 *	0.58 (0.52–0.65)	< 0.001 *	0.71 (0.45–1.12)	0.113	1.28 (1.14–1.44)	< 0.001 *
Hispanic	1.30 (1.12–1.51)	0.001 *	0.62 (0.43–0.90)	0.004 *	1.19 (0.53–2.65)	0.693	0.90 (0.80–1.01)	0.058	0.49 (0.26–0.93)	0.007 *	1.22 (1.06–1.41)	0.008 *
Other	1.48 (1.14–1.91)	0.009 *	1.27 (0.76–2.10)	0.403	0.60 (0.08–4.48)	0.535	0.93 (0.76–1.15)	0.502	1.09 (0.47–2.53)	0.838	0.99 (0.75–1.31)	0.952
VS White (Ref)												
*Disability*												
Not Disabled VS Disabled (Ref)	1.92 (1.43–2.57)	< 0.001 *	2.33 (1.26–4.30)	0.004 *	3.58 (0.77–16.62)	0.063	1.74 (1.43–2.11)	< 0.001 *	1.06 (0.44–2.53)	0.894	1.02 (0.81–1.28)	0.892

Education appeared to have a similar prescription pattern to income; patients with higher levels of education (Bachelor’s degree or higher) are more likely to receive ciclopirox (OR 1.64 (1.46–1.84), p < 0.001), efinaconazole (OR 3.07 (2.38–3.96), p < 0.001), tavaborole (OR 3.05 (1.48–6.30), p = 0.004), terbinafine (OR 1.36 (1.25–1.47), p < 0.001) and itraconazole (OR 2.08 (1.44–3.00), p < 0.001), but less likely to receive fluconazole (OR 0.82 (0.73–0.91), p = < 0.001). Table 3 summarizes the above results.

**Table 3:** Summary of findings for prescribed medications.

	Ciclopirox	Efinaconazole	Tavaborole	Terbinafine (oral)	Itraconazole	Fluconazole
*Age*	No difference	No difference	No difference	Younger patients more likely	Younger patients more likely	Younger patients more likely
*Sex at Birth*	Females more likely	Females more likely	Females more likely	No difference	No difference	Females more likely
*Income*	Higher income more likely	Higher income more likely	Higher income more likely	Higher income more likely	Higher income more likely	Lower income more likely
*Education*	Higher education more likely	Higher education more likely	Higher education more likely	Higher education more likely	Higher education more likely	Lower education more likely
*Race/Ethnicity*	White more likely than Black; Hispanic more likely than White	White more likely than Black; White more likely than Hispanic	White more likely than Black	White more likely than Black	White more likely than Hispanic	Black more likely than White; Hispanic more likely than White
*Disability*	More likely in able-bodied	More likely in able-bodied	No difference	More likely in able-bodied	No difference	No difference

Differences could also be observed between different race/ethnicity. Patients that identified as Black were less likely to be prescribed ciclopirox (OR 0.71 (0.91–0.83), p < 0.001), efinaconazole (OR 0.38 (0.26–0.56), p < 0.001), tavaborole (OR 0.09 (0.01–0.69), p < 0.001) and terbinafine (OR 0.58 (0.52–0.65), p < 0.001), but more likely to receive fluconazole (OR 1.28 (1.14–1.44), p < 0.001) compared to Whites.

### Surgical procedure trends

**Fig 5:**
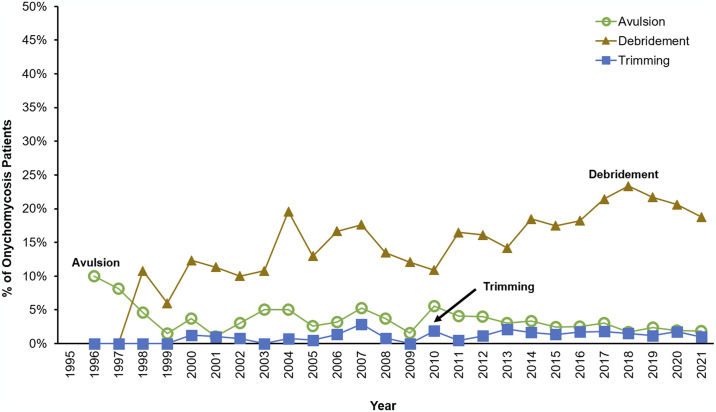
Percentage of onychomycosis patients undergoing nail related procedures per year.

There is a higher level of debridement compared to other procedures over time (Fig 5). The proportion of onychomycosis patients undergoing surgical procedures to treat onychomycosis increased with age (Fig 4B). Patients that were less than 60 years of age were more likely to undergo nail avulsion (OR 1.53 (1.21–1.93), p < 0.001) compared to those over 60 years of age (Table 4). Males were more likely to undergo debridement and trimming procedures when compared to females (OR 1.40 (1.16–1.69), p = 0.001; OR 2.22 (1.60–3.10), p < 0.001; respectively).

**Table 4:** Odds Ratios and P-values for surgical procedures.

	Nail Avulsion		Debridement of Nail(s)		Trimming of Nail(s)	
	OR	P-Value	OR	P-Value	OR	P-Value
*Geriatric*						
< 60 years VS > 60 years (Ref)	1.53 (1.21–1.93)	< 0.001 *	0.95 (0.81–1.12)	0.577	0.43 (0.33–0.55)	< 0.001 *
*Sex at Birth*						
Male Vs Female (Ref)	0.83 (0.66–1.05)	0.119	1.40 (1.16–1.69)	0.001 *	2.22 (1.60–3.10)	< 0.001 *
*Income*						
> $75,000/year VS < $75,000/year (Ref)	0.94 (0.76–1.18)	0.612	0.51 (0.41–0.63)	< 0.001 *	0.43 (0.30–0.62)	< 0.001 *
*Education*						
Bachelor’s Degree or Higher	0.95 (0.78–1.14)	0.561	0.71 (0.61–0.84)	< 0.001 *	0.62 (0.47–0.81)	< 0.001 *
VS Some College, High School or Less (Ref)						
*Race/Ethnicity*						
Black	0.78 (0.62–0.99)	0.033	1.19 (1.00–1.42)	0.058	0.41 (0.28–0.60)	< 0.001 *
Hispanic	0.79 (0.61–1.04)	0.076	0.77 (0.61–0.97)	0.018 *	0.35 (0.22–0.56)	< 0.001 *
Other	0.73 (0.43–1.23)	0.179	1.08 (0.75–14.58)	0.681	0.97 (0.55–1.72)	0.923
VS White (Ref)						
*Disability*						
Not Disabled VS Disabled (Ref)	0.79 (0.52–1.20)	0.278	0.50 (0.37–0.69)	<0.001 *	0.32 (0.18–0.55)	< 0.001 *
*Comorbidity*						
Tinea Pedis	1.92 (1.32–2.79)	< 0.001 *	6.06 (4.92–7.48)	<0.001 *	9.80 (3.05–31.50)	< 0.001 *
Obesity	2.13 (1.51–3.01)	< 0.001 *	6.33 (5.17–7.58)	<0.001 *	13.73 (4.37–43.07)	< 0.001 *
HIV	1.66 (0.93–2.97)	0.130	4.86 (3.71–6.36)	<0.001 *	18.10 (5.22–62.72)	< 0.001 *
Type 2 DM	1.82 (1.28–2.58)	< 0.001 *	7.55 (6.17–9.25)	<0.001 *	16.83 (5.37–52.78)	< 0.001 *
VS No Comorbidity (Ref)						

Patients with higher annual income (> $75,000 USD/year) were less likely to undergo debridement and trimming procedures (OR 0.51 (0.41–0.63), p < 0.001; OR 0.43 (0.30–0.62), p < 0.001; respectively). Higher levels of education showed a similar trend, with patients that held a Bachelor’s degree or higher being less likely to undergo debridement and trimming (OR 0.71 (0.61–0.84), p < 0.001; OR 0.62 (0.47–0.81), p = 0.003; respectively).

Nail avulsion was performed more frequently in patients with tinea pedis (OR 1.92 (1.32–2.79), p < 0.001), obesity (OR 2.13 (1.51–3.01), p < 0.001), but not in HIV (OR 1.66 (0.93–2.97), p = 0.130). Debridement of the nail or nails was performed more frequently in comorbid patients (OR ranging from 4.86 (3.71–6.36) for HIV (p < 0.001), to 7.55 (6.17–9.25) for type 2 DM (p < 0.001). Additionally, nail trimming occurred more frequently in comorbid compared to non-comorbid patients (OR ranging from 9.81 (3.05–31.50) in tinea pedis (p < 0.001), to 18.10 (5.22–62.72) in HIV (p < 0.001). Table 5 summarizes the above results.

**Table 5:** Summary of findings for surgical procedures.

	Nail Avulsion	Debridement of Nail(s)	Trimming of Nail(s)
*Age*	Younger more likely	No difference	Older more likely
*Sex at Birth*	No difference	Males more likely	Males more likely
*Income*	No difference	Lower income more likely	Lower income more likely
*Education*	No difference	Lower education more likely	Lower education more likely
*Race/Ethnicity*	No difference	White more likely than Hispanic	White more likely than Black; White more likely than Hispanic
*Disability*	No difference	Disabled more likely	Disabled more likely
*Tinea Pedis*	Comorbid more likely	Comorbid more likely	Comorbid more likely
*Obesity*	Comorbid more likely	Comorbid more likely	Comorbid more likely
*HIV*	No difference	Comorbid more likely	Comorbid more likely
*Type 2 Diabetes*	Comorbid more likely	Comorbid more likely	Comorbid more likely

## Discussion

Our analysis of available AoU data demonstrates clear differences in onychomycosis management between different populations. Building upon the work by Albucker et al. and Moseley et al. [15], our findings highlight challenging real-world scenarios in managing onychomycosis patients where this chronic condition is often seen in conjunction with other co-morbid illnesses, for example, diabetes. Furthermore, in view of a growing recognition of healthcare inequities [19, 20], we identified potential disparities in access to care for the management of onychomycosis—especially concerning minority groups and low-income individuals who are at a higher risk—where the ordering of diagnostic testing and prescribing of treatments were linked to socioeconomic factors.

In agreement with previous studies, onychomycosis was most prevalent in those over 60 years of age [2, 14, 15]. The burden was also 20% higher in those who had lower income (< $75,000/year), and in Black patients compared to White patients [14, 15]. We have presented an extensive list of comorbidities associated with a higher burden of onychomycosis which expands on previous work [9, 14, 21, 22]. We introduce the concept of ‘onychomycotic foot’ where certain co-morbidities are more likely to be associated with onychomycosis as shown in Fig 2. A more than 30-times higher risk was observed in tinea pedis patients, which is one of the most common clinical observations in onychomycosis patients [23]. A co-occurrence of tinea pedis may indicate the initial site of infection; treatment of tinea pedis is currently recommended to prevent the recurrence and spread of onychomycosis [23]. The higher risk for onychomycosis (OR: 12.9) observed in patients with lower limb deformities mirrors that observed in patients with peripheral venous insufficiency (OR: 12.3) and peripheral arterial disease (OR: 11.3). Venous insufficiency can be associated with skin and nail alternations including ulcerations and dystrophic nails (e.g., onychogryphosis)–often characterized by nail plate thickening, brittleness or discoloration—that creates a point of entry for dermatophytic pathogens [24]. Similarly, a higher risk in patients with peripheral arterial disease could be due to reduced oxygenation and nutrient exchange in the foot leading to skin and nail alternations, which can be exacerbated by smoking according to a previous study [25].

In the literature there are conflicting reports about the association between onychomycosis and psoriasis [26]; however, this study supports this association with a 7.6-times higher likelihood that could be attributed to use of immunosuppressive medications, dystrophic nails changes and abnormal capillaries. The association between diabetes and onychomycosis has been well established due to poor peripheral circulation that predisposes this patient population to higher risks of secondary infections and ulceration [9, 27]; development of diabetic foot ulcers is exacerbated in the presence of onychomycosis which may lead to amputations and mortality [9, 28, 29].

Despite the well-established risk of misdiagnosis at the point-of-care [30] (i.e., 50% of clinically suspected cases test negative on mycology), diagnostic testing remains a significant challenge in providing quality care for onychomycosis patients. Previous studies have consistently demonstrated that diagnostic testing is offered to only one-quarter of patients or less when there is a clinical suspicion of onychomycosis [31]. As well, our finding suggests that certain high-risk groups (i.e. patients with tinea pedis or obesity) were given priority for testing; however, the reverse trend was observed for social minority groups. In particular, Blacks and Hispanics—who are at a higher risk of contracting onychomycosis—were about 50% less likely to get tested than Whites [15]. Persons with lower incomes or without a Bachelor’s degree were also less likely to receive testing despite of the higher risks in developing onychomycosis. These disparities may be linked to the lack of specialist clinics in rural areas where 88% of residents have no access to a dermatologist [32]. In one U.S. survey, <50% of primary care providers reported being comfortable with diagnosing and treating skin of color [33]. The lack of diagnostic testing is an ongoing issue that extends beyond the U.S [34, 35]. Further investigations and renewed calls for improving the global management of onychomycosis are warranted.

There are clear differences in which patients receive prescription medications. Oral medications are more likely to be prescribed to younger patients. This could be attributed to an increasing comorbidity burden among the elderly, where drug interactions could be a concern, as cytochrome P450 enzymes limit the use of systemic antifungal agents; [36] additionally, older subjects may have reduced hepatic reserve. Topical agents, typically requiring long-term compliance were more frequently prescribed for females. This patient group is more vulnerable to the psychological burdens associated with onychomycosis [37], and may be more receptive to applying topical agents that typically require long term application [38].

Individuals with lower incomes, without a Bachelor’s degree, Blacks and Hispanics, as well as females, were more frequently observed to be prescribed fluconazole. This triazole is approved in some European countries for onychomycosis treatment [39], and is frequently prescribed off-label in the US [40, 41]. The availability of fluconazole as a generic medication has greatly reduced its costs (14.6% reduction from 2013–2018), making it more accessible across patient groups [42]. Additionally, as fluconazole is approved for treating vaginal candidiasis [43], the physician may prescribe it for this indication in patients with concomitant onychomycosis. Newer medications (i.e., efinaconazole, tavaborole), with high monthly costs ($807–1392 USD) often requiring prior authorization for insurance coverage, were significantly less prescribed for social minority groups [40, 43–49].

In patients with comorbidities, we found that almost all nail procedures were more likely to be performed possibly due to concerns of drug-drug interactions that favors non-pharmacological interventions [36]. The frequency of these procedures increases with age, which is likely due to older patients who may have more difficulty caring for their nails. Debridement was found to be performed more frequently than avulsion or trimming. The majority of onychomycosis case are initially diagnosed by podiatry, followed by general practitioners and dermatologists, with podiatrists generally more likely to perform debridement procedures as the first step to managing onychomycosis [31]. Lower income individuals and those with lower education were more likely to undergo nail trimming and debridement procedures in the clinic.

While the All of Us research program provides novel insights on the current state of onychomycosis management in the US, these findings may have some applicability to the global patient population at large. Due to the voluntary nature of data reporting, our work is subjected to sampling and recall biases. Cai et al. examined the accuracy of EHR data within the AoU dataset, finding an overall incongruence rate of 0.86% for sex specific conditions [50], with the actual rate of error in EHR data potentially being higher when all conditions are considered. These types of inaccuracies could be due to data entry errors [51]. Even if the incongruency rate is small, this could still potentially skew results, particularly when examining relatively rare conditions [50]. Another limitation of the AoU is that there is limited information on the location and specialty of practice, insurance coverage, and availability of diagnostic testing results within the AoU dataset, which are potential confounding variables.

## Conclusions

In this cross-sectional study of 266,612 individuals, including 18,763 onychomycosis patients, participating in the All of Us research program, we identified a significant comorbidity burden that complicates the management of this chronic, slow-progressive infection. This finding is especially concerning for social minority groups who were found to have a higher risk of contracting onychomycosis. Despite continued advocacy efforts, significant disparities exist in the current testing and treatment practices. Actionable strategies such as expanding healthcare coverage, reducing cross-cultural communication barriers and increased educational efforts, are needed [19].

## Supporting information

S1 Table Number of participants in comorbid and control groups. (DOCX)

S2 Table Number of participants in each demographic group. (DOCX)

S3 Table Conditions, diagnostic testing, medical and surgical concept ID codes. (DOCX)

S1 File STOBE/RECORD checklists. (DOCX)

S2 File Human subject in research checklist. (DOCX)
