# Supplementary material for: Epidemiological trends and healthcare disparities in onychomycosis: An analysis of the All of Us research program
Source: PLoS One. 2025 Jan 14;20(1):e0316681. doi: 10.1371/journal.pone.0316681 (PMC11731872; doi:10.1371/journal.pone.0316681)
Supplement: S1 Table — (DOCX) [file pone.0316681.s001.docx]

**S1 Table** – Number of participants in comorbid and control groups

| **Comorbidity** | **# Comorbid Patients** | **# Controls** |
| --- | --- | --- |
| Diabetes | 55623 | 78417 |
| Obesity | 76409 | 95516 |
| Tinea Pedis | 9135 | 21392 |
| Peripheral Artery Disease | 5642 | 12617 |
| Peripheral Venous Insufficiency | 10616 | 23215 |
| Peripheral Neuropathy in Lower Limbs | 34531 | 60423 |
| Edema in Lower Limbs | 352 | 860 |
| Arthritis in Lower Limbs | 10317 | 24623 |
| Deformities of the Lower Limbs | 24937 | 47917 |
| Hemodialysis | 338 | 910 |
| Renal Transplant | 2203 | 5974 |
| HIV positive | 5188 | 12854 |
| Psoriasis | 7377 | 18543 |
| Lupus Erythematosus | 3964 | 11996 |
| Chronic Liver Disease | 16990 | 36955 |
