# Supplementary material for: Epidemiological trends and healthcare disparities in onychomycosis: An analysis of the All of Us research program
Source: PLoS One. 2025 Jan 14;20(1):e0316681. doi: 10.1371/journal.pone.0316681 (PMC11731872; doi:10.1371/journal.pone.0316681)
Supplement: S2 Table — (DOCX) [file pone.0316681.s002.docx]

**S2 Table –** Number of participants in each demographic group

| **Factor** | |  | **Number of participants** |
| --- | --- | --- | --- |
| ***Age at onychomycosis diagnosis***  ***or***  ***Age at program intake (no onychomycosis)*** | |  |  |
|  | **Less than 18 years** |  | 1313 |
|  | **Greater than 60 years** |  | 98356 |
| ***Economic*** | |  |  |
|  | **< $75,000/year** |  | 135372 |
|  | **> $75,000/year** |  | 74693 |
| ***Education*** | |  |  |
|  | **Less than Bachelor’s degree** |  | 145942 |
|  | **Bachelor’s degree or higher** |  | 112322 |
| ***Ethnicity/Race*** | |  |  |
|  | **White** |  | 142220 |
|  | **Black** |  | 50088 |
|  | **Hispanic** |  | 45693 |
|  | **Other** |  | 20263 |
