# Supplementary material for: Epidemiological trends and healthcare disparities in onychomycosis: An analysis of the All of Us research program
Source: PLoS One. 2025 Jan 14;20(1):e0316681. doi: 10.1371/journal.pone.0316681 (PMC11731872; doi:10.1371/journal.pone.0316681)
Supplement: S3 Table — (DOCX) [file pone.0316681.s003.docx]

**Table S3** – Conditions, diagnostic testing, medical and surgical concept ID codes.

|  | **Concept name** | **Concept ID** |
| --- | --- | --- |
|  |  |  |
| *Conditions* |  |  |
|  |  |  |
|  | Onychomycosis due to dermatophyte | 140648 |
|  | Type 2 Diabetes Mellitus | 201826 |
|  | Obesity | 433736 |
|  | Tinea Pedis | 133141 |
|  | Peripheral Artery Disease | 3654996 |
|  | Peripheral Venous Insufficiency | 321596 |
|  | Peripheral Neuropathy in Lower Limbs | 43531034 |
|  | Edema in Lower Limbs | 42709835 |
|  | Arthritis in Lower Limbs | 4094283 |
|  | Deformities of the Lower Limbs | 42709843 |
|  | HIV Positive | 439727 |
|  | Psoriasis | 140168 |
|  | Lupus Erythematosus | 255891 |
|  | Chronic Liver Disease | 4212540 |
|  |  |  |
| *Non-nail related procedures* | |  |
|  |  |  |
|  | Hemodialysis | 4120120 |
|  | Renal Transplant | 4322471 |
|  |  |  |
| *Nail related procedures* | |  |
|  | |  |
|  | Nail Avulsion | 2102079 |
|  |  | 2102078 |
|  |  | 2722193 |
|  | Debridement of Nail(s) | 2006538 |
|  |  | 2102076 |
|  |  | 2102077 |
|  | Trimming of Nail(s) | 2617229 |
|  |  |  |
|  |  |  |
| *Prescription Medications* | |  |
|  | |  |
|  | Ciclopirox | 950133 |
|  |  | 19075343 |
|  |  | 19135270 |
|  |  | 40238087 |
|  |  | 42707838 |
|  |  |  |
|  | Efinaconazole | 45775080 |
|  |  | 45775083 |
|  |  | 45775087 |
|  |  |  |
|  | Tavaborole | 45776537 |
|  |  | 45776540 |
|  |  | 45776544 |
|  |  |  |
|  | Terbinafine | 1741309 |
|  |  | 1741402 |
|  |  | 19006863 |
|  |  | 19083463 |
|  |  | 19116141 |
|  |  | 40171255 |
|  |  |  |
|  | Itraconazole | 1703653 |
|  |  | 1703661 |
|  |  | 19078728 |
|  |  | 40184844 |
|  |  | 40184845 |
|  |  | 42707244 |
|  |  |  |
|  | Fluconazole | 1754995 |
|  |  | 1754996 |
|  |  | 1755073 |
|  |  | 1755074 |
|  |  | 1755076 |
|  |  | 1755077 |
|  |  | 19019003 |
|  |  | 19019004 |
|  |  | 19116140 |
|  |  | 19117680 |
|  |  | 19118266 |
|  |  |  |
| *Diagnostic Testing* | |  |
|  | Antimicrobial susceptibility test | 4032801 |
|  | Candida sp DNA [Presence] in Specimen by NAA with probe detection | 1175462 |
|  | Fungal culture, with definitive identification | 4015193 |
|  | Fungus [Presence] in Nail by KOH preparation | 40758435 |
|  | Fungus [Presence] in Specimen by KOH preparation | 40758434 |
|  | Fungus identified in Nail by Culture | 3005433 |
|  | Fungus identified in Skin by Culture | 3002951 |
|  | Fungus identified \| Nail \| Microbiology | 37055429 |
|  | Fungus identified \| XXX \| Microbiology | 37038886 |
|  | Microbiologic smear with interpretation, wet mount with simple stain | 4014503 |
|  | Mycology culture | 4165398 |
|  | Yeast [Presence] in Nail by KOH preparation | 40758443 |
|  | Yeast culture | 4046245 |
|  | Yeast.pseudohyphae [Presence] in Nail by KOH preparation | 40758439 |
|  | Microscopic observation [Identifier] in Nail by KOH preparation | 3020394 |
